# A Comparative Overview of the Flagellar Apparatus of Dinoflagellate, Perkinsids and Colpodellids

**DOI:** 10.3390/microorganisms2010073

**Published:** 2014-03-10

**Authors:** Noriko Okamoto, Patrick J. Keeling

**Affiliations:** Centre for Microbial Diversity and Evolution, Department of Botany, University of British Columbia, 3529-6270 University Boulevard, Vancouver, BC V6T 1Z4, Canada; E-Mail: okamoton@mail.ubc.ca

**Keywords:** apicomplexa, *Chromera*, *Colpodella*, colpodellids, dinoflagellates, Dinozoa, *Oxyrrhis*, perkinsids, *Perkinsus*, *Psammosa*, *Rastrimonas*, the apical complex, the flagellar apparatus, *Vitrella*

## Abstract

Dinoflagellates are a member of the Alveolata, and elucidation of the early evolution of alveolates is important for our understanding of dinoflagellates, and *vice versa.* The ultrastructure of the flagellar apparatus has been described from several dinoflagellates in the last few decades, and the basic components appear to be well conserved. The typical dinoflagellate apparatus is composed of two basal bodies surrounded by striated collars attached to a connective fiber. The longitudinal basal body is connected to a longitudinal microtubular root (LMR; equivalent of R1) and single microtubular root (R2), whereas the transverse basal body is connected to a transverse microtubular root (TMR; R3) and transverse striated root (TSR) with a microtubule (R4). Some of these components, especially the connective fibers and collars, are dinoflagellate specific characteristics that make their flagellar apparatus relatively complex. We also compare these structures with the flagellar apparatus from a number of close relatives of dinoflagellates and their sister, the apicomplexans, including colpodellids, perkinsids, and *Psammosa*. Though the ultrastructural knowledge of these lineages is still relatively modest, it provides us with an interesting viewpoint of the character evolution of the flagellar apparatus among those lineages.

## 1. Introduction

Dinoflagellates are one of the most abundant protist groups in aquatic environments, and play important roles as primary producers, grazers, and parasites [1,2,3,4,5]. Extant dinoflagellates, especially the dinokaryotes, or the core dinoflagellates, are distinct from any other protists in both morphology (dinokaryon, heteromorphic flagella, the cell architecture with epicone and hypocone) and genomic features (large genomes, condensed chromatin, trans-spliced mRNAs). We have learned a great deal about the evolution of some of these features by comparing dinoflagellates to their closest relatives. According to the current view based on molecular phylogenies, the closest sister group to dinoflagellates is the perkinsids [6,7,8,9,10,11,12,13,14,15,16,17]. Perkinsids are marine protists consisting of the parasitic genera *Perkinsus* and *Parvilucifera*, and the predatory *Rastrimonas* [6]. Together, the dinoflagellates and perkinsids have been classified as Dinozoa, one of three major groups in Alveolata. Dinozoa is sister to the parasitic Apicomplexa that includes human parasites such as, *Plasmodium* (the malaria parasites), *Babesia*, *Toxoplasm*a, and *Cryptosporidium*; or parasites of cattle or poultry including *Theileria*, *Eimeria*, and *Neospora*. There are several lineages related to the apicomplexans, such as the photosynthetic genera *Chromera* and *Vitrella*, and free-living predators known as colpodellids [6,18,19,20,21,22,23,24]. 

Inferring dinoflagellate character evolution in the context of the Alveolata is challenging. Not only have dinoflagellates undergone several distinctive changes, but the other lineages have also developed their own unique features. As all three major groups are important organisms in their own ways, ultrastructural investigations on each have been conducted since the 1960s. However, because they were largely done in isolation, there is significant confusion in terminology, since major ultrastructural discoveries were made before the molecular phylogeny revealed the relationships between the organisms. The recent leap in our understanding of genomic, transcriptomic, and proteomic information means that we now have considerable molecular data from the apicomplexans, ciliates, and dinoflagellates, which altogether enables us to compare these distant lineages (although the availability of such data from the “intermediate” lineages like perkinsids and colpodellids remains a problem). In this review, we will compare the cytoskeletal element, especially the flagellar apparatus and the feeding structures, of dinoflagellates and their nearest relatives to begin to construct a fundamental understanding of the evolution of these structures.

## 2. Diversity of the Flagellar Apparatus of the Dinoflagellates and Related Lineages

### 2.1. Flagellar Apparatus of Dinoflagellates

#### 2.1.1. Dinokaryotes

A typical dinoflagellate cell in a motile stage has two flagella. Flagellar configuration is traditionally classified into three categories; dinokont (most of the dinokaryotes), desmokont (e.g., prorocentrids) and opisthokont (e.g., *Oxyrrhis marina*) according to the position of the flagellar insertion in relation to the cell and transverse groove, if present. Regardless of the type of flagellar configurations, the dinoflagellate flagellar apparatus retains unity among those different groups. For instance, a desmokont flagellar apparatus is a variation of dinokont that has an extreme basal body angle [25].

Electron microscopic studies of dinoflagellate flagellar apparatus first appeared in the 1960s and have continued. Earlier reports emphasized the ultrastructure of flagella and basal bodies [26,27], but a few studies reported the whole complex flagellar apparatus [28]. Several syntheses of these data have already been reported, for example an early review by Moestrup [28], a comprehensive review of the basic characters of dinoflagellate flagellar apparatus by Roberts [29], the terminology of which was later standardized across the group as a whole [30]. The most recent exhaustive review of the dinoflagellate flagellar apparatus is by Calado [31]. 

**Figure 1:**
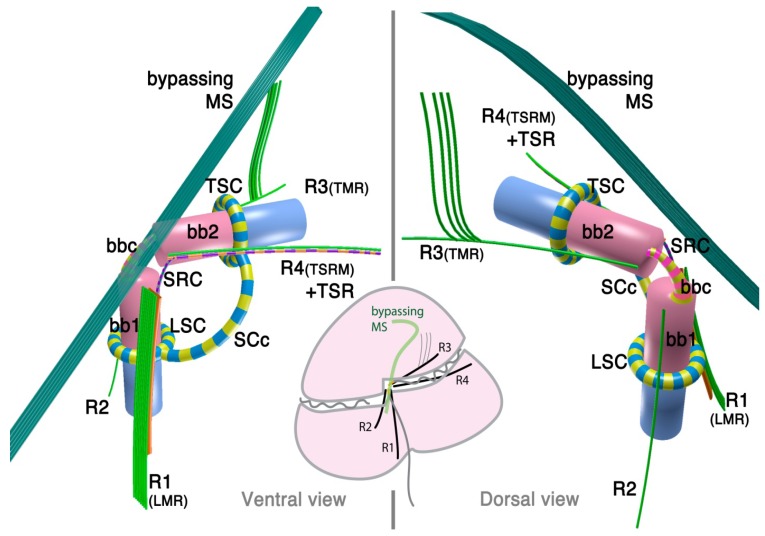
Flagellar apparatus of dinokaryotes. Figure is drawn after [30,31]. bb1= basal body 1 (longitudinal basal body), bb2 = basal body 2 (transverse basal body), bbc = basal body connective (for different terminology, see the text), LSC = longitudinal striated collar, MS = microtubular strands, SCc = striated collar connective, SRC = striated root connective (between R1 and TSR), TMR = transverse microtubular root, TSC = transverse striated collar, TSR = transverse striated collar, R1 = root 1 (or LMR), R2 = root 2, R3 = root 3, R4 = root 4.

The dinoflagellate flagellar apparatus is composed of two basal bodies, three or four microtubular roots, and connective fibers (Figure 1). The angle of the basal bodies varies between and within a given family. It is typically around 90° or more, with some extreme exceptions being almost parallel (0°) to counter-parallel (180°). The longitudinal basal body is older (hence, basal body 1: BB1) and the transverse basal body is younger (hence, basal body 2: BB2) [32].

Most species examined so far possess three microtubular roots, namely, R1 (historically referred to as Longitudinal Microtubular Root; LMR), R3 (or Transverse Microtubular Root; TMR) and R4 (Transverse Striated Root associated Microtubule; TSRM) that are associated with transverse striated root (TSR). R2 is generally a single microtubular root when present, although it is often missing. 

The dinoflagellate flagellar apparatus also includes various fibrous structures, which lend considerable morphological complexity to the system. Sometimes these fibrous structures join the basal bodies themselves, with or without an alternating striation pattern. The connecting fibers are called “layered connective” (LC) in peridinioids [31], striated basal body connective (SBC) in gonyaulacoids [33], basal body connective (BBC) in some species of Tovelliaceae [25] and Suessiaceae [34], and cross-banded fiber (CBF) [35]. Albeit widespread, the inter-basal body connective is not always present. In contrast, another striated connective (SRC) between R1 (LMR) and R4 (or TSR associated with R4) is universally present in the species investigated to date [31]. Besides SRC, R1 (LMR) is often connected to either BB1 or BB2 via another dark-stained connective fiber. 

In addition to the already complex flagellar apparatus, the flagellar canals of both flagella are often lined with a striated fibrous structure that forms either a complete or incomplete “collar”. Those collars are named Transverse or Longitudinal Striated Collars (TSC and LSC). The TSC and LSC are interconnected by an extension of the collars (striated collar connective; SCc) or an additional fibrous structure (accessory striated collar connective; ASCc). Sometimes the TSC/LSC/SCc complex is further associated to ventral ridge (vr) fibers. TSC/LSC/SCc probably contains centrin or its homologue in *Akashiwo sanguinea* [36].

Although it is typically not considered to be part of flagellar apparatus, there is also a bundle of numerous microtubules (typically 20 or more) that bypass close to the flagellar apparatus in many dinoflagellates, often referred to as the microtubular strand or basket (MS or MB). Usually, the MS consists of a single layer, though it can be a multilayered bundle in some species [37]. The MS is often associated with electron dense vesicles and has been confirmed to be a part of the peduncle (a type of feeding apparatus used for myzocytosis) in some species of peridinioids, Tovelliaceae, Borghiellaceae, Suessiaceae and Pfiesteriaceae; whereas in gonyaulacoids, the analogous structure has not been shown to extend into the peduncle. Often, MS associated with the peduncle is also connected to TSC/LSC/SCc complex via a fibrous structure connected near the emerging point of the peduncle.

#### 2.1.2. *Oxyrrhis marina*: A Basal Lineage of Dinoflagellates

*Oxyrrhis marina* is a free-living heterotrophic flagellate, often found in coastal waters and tide pools, where it feeds on smaller algae. Since *O. marina* is one of the more easily cultivatable grazers and has been used as a model for heterotrophic processes, its ultrastructural and genetic features are extensively studied. Molecular data group *O. marina* with the dinoflagellates, but it lacks many typical dinoflagellate morphological features, including the basic epicone-hypocone configuration, the dinokaryon, or typical dinoflagellate trichocysts. The flagellar apparatus, however, has a striking resemblance to that of core dinoflagellates. 

**Figure 2:**
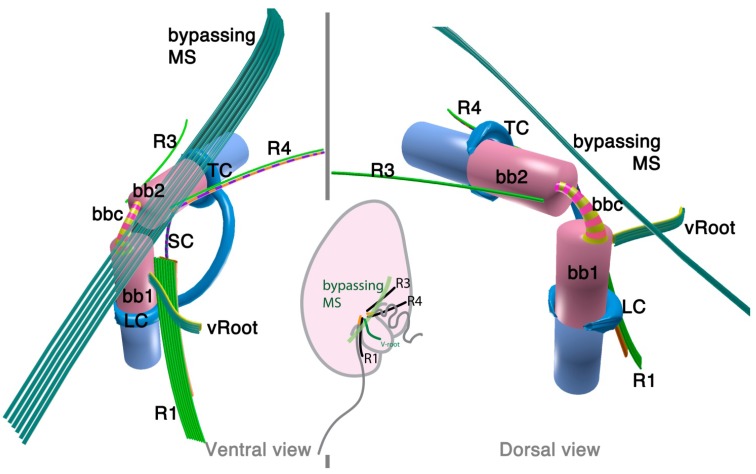
Flagellar apparatus of *Oxyrrhis marina.* Figure is drawn after [38,39,40]. Abbreviations are the same as in Figure 1. LC = longitudinal collar, TC = transverse collar, vRoot = ventral ridge root.

The 3D reconstruction of the flagellar apparatus of *O. marina* was first examined by Dodge and Crawford [38], then by Roberts [39]. The majority of the structures, especially the configuration of microtubular roots and connecting fibers, are well understood owing to these studies. Figure 2 illustrates the flagellar apparatus of *O. marina*. It has three microtubular roots (R1, R3 and R4). R2 has not been recognized and most likely is absent, as is true with some dinokaryotes. 

Besides the standard microtubular roots, the flagellar apparatus of *O. marina* has two distinct microtubular structures, namely, the ventral microtubular root (VR) and ventral ridge microtubules (VRM; is also referred to as transverse microtubular band or TMB) [36,40]. VR is formed by 3–4 longitudinally aligned microtubules, associated with an electron dense material on the left side. VR emerges from the proximal ventral face of the longitudinal basal body and extends into the bulge called “tentacle”. VRM/TMB run almost transverse to the cell and form the ventral ridge. It is also associated with fibrous material (ventral ridge fiber: VRF) on its proximal/dorsal side near the flagellar apparatus. The VRF is connected to electron dense half-rings associated with the transverse and longitudinal basal body [36,40]. Interestingly, VRF and the half-rings contain centrin or its homologue [40]. This indicates that these fibers are most likely homologous to the LSC-TSC-SCC complex, which has also been demonstrated to react to anti-centrin antibodies [29]. In some dinokaryotes, this fibrous complex connects the flagellar apparatus to the microtubular basket that forms the peduncle. Although *O. marina* feeds by phagocytosis and not myzocytosis (which is mediated by the peduncle), the role of the VRF could be analogous in that it defines the feeding structure during the phagocytosis. It is tempting to speculate that the VRF may be homologous to the microtubular component of the peduncle.

#### 2.1.3. *Psammosa pacifica*

**Figure 3:**
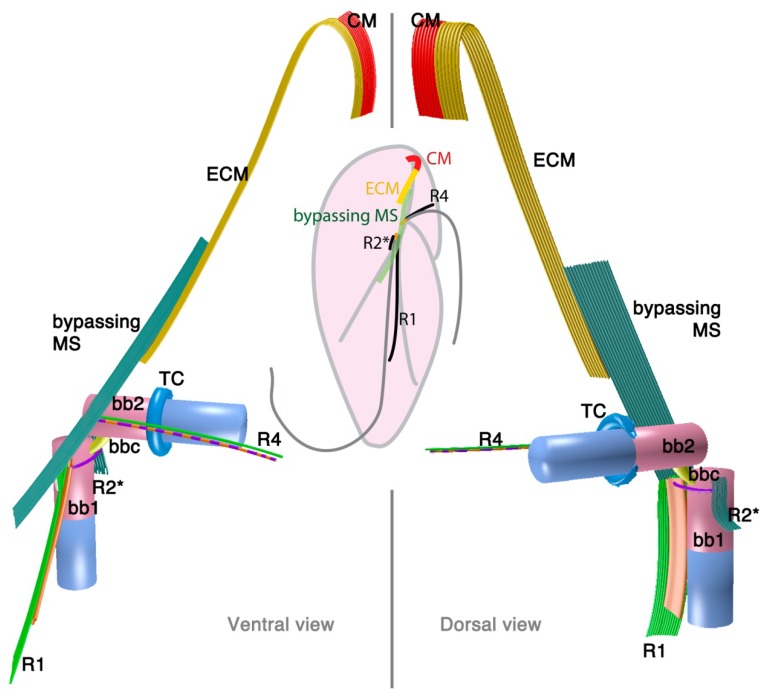
Flagellar apparatus and the microtubular skeleton of the apical complex of *Psammosa pacifica.* Figure is drawn after [41]. Abbreviations are the same as in Figure 1 and Figure 2. CM = conoid microtubules, ECM = extended conoid microtubules.

*Psammosa pacifica* is also a free-living heterotrophic flagellate that branches at the base of the dinoflagellates, apparently before *Oxyrrhis* [8]. Interestingly, this species has been found to contain an apical complex, the structure for which apicomplexans are named, and which is also found in colpodellids and perkinsids. The flagellar apparatus of the *Psammosa pacifica* has R1, putative R2, and R4, as well as a bypassing microtubular strand that ultimately connects conoid microtubules (CM) to the apical complex via extended conoid microtubules (ECM) [41] (Figure 3). R1 is made up of multiple microtubules and lined with a layered sheet on the left side. The putative R2 is a very short 6-microtubular structure on the dorsal side of BB1. This microtubular structure is distinct from the typical R2 in dinoflagellates, which is a long single microtubule. R3 is missing. R4 is a typical single-microtubular root accompanied by transverse striated fiber (TSR). The two basal bodies are interconnected by multiple small connective fibers that also attach to R1 and the putative R2.

### 2.2. Perkinsids: The Sister Group of the Dinoflagellates

Perkinsids are the immediate sister group of the dinoflagellates (assuming one includes *Oxyrrhis* and *Pasammosa* in the dinoflagellates, which is questionable given their position in the tree and morphology). The majority of currently known perkinsids are parasitic, such as *Perkinsus* and *Parvilucifera*; though it also includes free-living grazers such as *Rastrimonas*. The parasitic lineages typically have a flagellate stage for dispersal and infection. The ultrastructure of the flagellar apparatus is known from two species, *Parvilucifera infectans* [42] and *Rastrimonas subtilis* (= *Cryptophagus subtilis*) [43].

#### 2.2.1. *Parvilucifera infectans*

**Figure 4:**
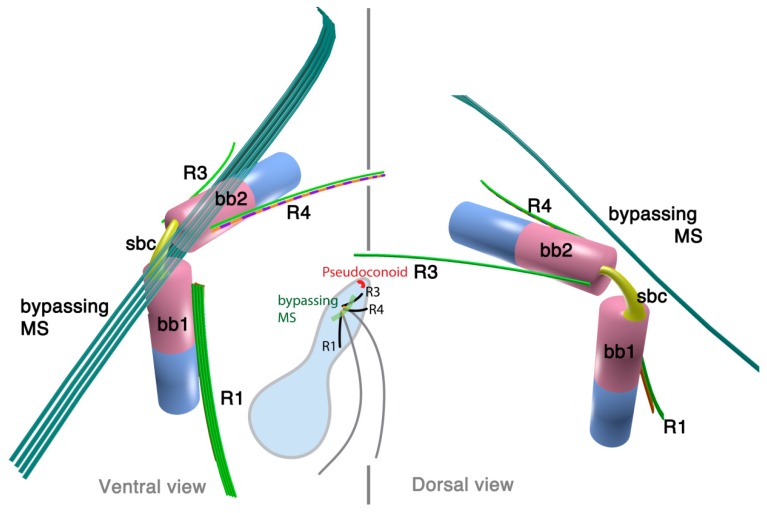
Flagellar apparatus of *Parvilucifera infectans.* Figure is drawn after [42]. Abbreviation is the same as in Figure 1 and Figure 2.

*Parvilucifera* is a parasite of various dinoflagellates such as *Dinophysis*, *Alexandrium*, and *Karenia*. A flagellate zoospore infects a host cell, where it develops a round body (sporangium) and produces dozens of zoospores. Once matured, sporangium forms a germ tube to release the zoospores [42,44]. A zoospore of *P. infectans* has two flagella emerging near the anterior apex of the cell. At the insertion point, basal bodies are arranged at right angles, and are connected with fibrous structures; namely, striated basal connective (SBC) and the inner and the outer fibers (Figure 4). The microtubular roots share much in common with the dinoflagellates; R1, R3 and R4. R1 is composed of four microtubules and runs longitudinally at the ventral side of the longitudinal basal body, lined with a “boxlike” structure that connects to the basal body with a thin strand. Both R3 and R4 are single microtubular roots, with R4 associated with the transverse striated fiber. Besides the microtubular roots, *P. infectans* also possesses a set of 4–5 microtubules bypassing the longitudinal basal body. There is neither an obvious fibrous structure associated with these bypassing microtubules, nor is there a “collar” associated with the basal bodies, unlike those found in dinoflagellates including *O. marina*.

#### 2.2.2. *Rastrimonas subtilis*

*Rastrimonas subtilis* (*Cryptophagus subtilis* in the original description [43,45]) is a parasite of cryptophytes (microalgae), which is classified as a perkinsid, although there is no molecular data that are available to date to confirm this. The life cycle of *R. subtilis* somewhat resembles that of *Parvilucifera*, though without developing a “sporangium” or any surrounding membrane. The infectious stage has two flagella that emerge sub-apically from two basal bodies that are arranged at right angles and interconnected by fibrous structures (“arms” and “microfibrils”). There is no report of a fibrous structure that resembles “collars”. Four microtubular roots form the flagellar apparatus, namely, R1, R2, R3 and R4 (note the numbering used in the description has been altered here for consistency with Moestrup 2000). All the roots are posteriorly extending from the sub-apical basal bodies. R1 is composed of 4–5 microtubules attached to the ventral side of the longitudinal basal body (BB1) and is lined with a “dense lamina” on the distal side facing the plasma membrane (and lacking any other fibrous connective structures, unlike *P. infectans* and dinoflagellates). R2 is composed of two microtubules positioned on the dorsal side of BB1. R3 is composed of 3–4 microtubules on the dorsal side of the transverse basal body (BB2), and is associated with a “dense lamina” as is R1. R4 is composed of two microtubules on the ventral side of the BB2, and is associated with a dense fiber on its proximal side.

### 2.3. Colpodellids: Sisters of the Apicomplexan Parasites

On the other side of the split between dinoflagellates and apicomplexans is another lineage of importance, a collection of close relatives of the apicomplexans collectively referred to as colpodellids. Traditionally, colpodellids are described as predatory or sometimes as “ectoparasitic”, where several cells attach to a large prey cell, such as a ciliate, and ingest the cytoplasm from outside. The genus *Colpodella* was recently reviewed and amended based mainly on morphological characters [44], but this classification scheme has not been thoroughly tested with molecular data. Colpodellids are most likely para- or poly-phyletic based on a handful of colpodellid sequences and other environmental sequences available to date [19,21,46,47]. Indeed, relatives of colpodellids include photosynthetic lineages such as *Chromera* [20], *Vitrella* [21] and probably many more that are undescribed [19], as well as an infectious agent to an immuno-compromised human [47]. 

Ultrastructural studies have been conducted on a number of species of *Colpodella sensu*, Simpson & Patterson [48,49,50,51,52,53]. Some of them have features shared with dinoflagellates, which is another reason to suspect there are taxonomical issues with colpodellids. Unfortunately, there is no molecular information from species for which ultrastructural data are also available. This severely impairs our ability to understand the character evolution or infer the ancestral state of the common ancestor of Myzozoa. Further studies that elucidate both morphology and the molecular phylogenetic position of the same species would greatly aid our understanding of Myzozoan evolution. Meanwhile in this review, we will focus on three species that are relatively well described at the ultrastructural level, *C. vorax* [48], *C. gonderi* [49] and *C. perforans* [52], without worrying too much about their exact position in the tree.

#### 2.3.1. *Colpodella vorax*

**Figure 5:**
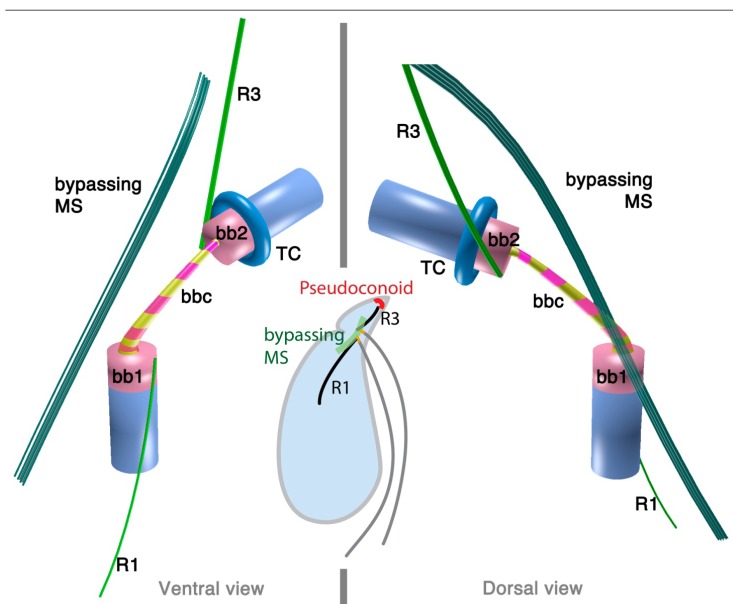
Flagellar apparatus of *Colpodella vorax* Figure is drawn after [48]. For detailed explanation, see text. Abbreviations are the same as in Figure 1 and Figure 2.

*Colpodella vorax* is a free-living heterotrophic flagellate that preys on other flagellates such as *Bodo caudatus* [48]. The two flagella are inserted sub-apically posterior to the rostrum. Basal bodies are arranged at about 120° to each other (Figure 5). The basal bodies are noticeably short and distant from each other (0.8 µm). They are interconnected with striated connective material (referred to as “plurilamellar structure”). The transverse basal body (BB2) has a collar.

There are three microtubular structures associated to the flagellar apparatus: R1, R3/4 and a bypassing microtubular strand. The posterior basal body (BB1) is associated with R1, a 2–3 microtubular root that is short in length, emerging from the ventral side of BB1 and extending posteriorly. The anterior basal body (BB2) is associated with a three-microtubular root (“anterior root” in the original description) which extends along the anterior flagellar pit towards the apex, although not actually demonstrably merging with the apical complex. It is not clear if this root is R3 or R4. The anterior flagellar pit also has a fibrous lining, which is possibly homologous to the flagellar collar of dinoflagellates. The bypassing 6–7 microtubules, which are referred to as the oblique root (oR) in the original report, emerge from the dorsal region of the BB1, passing over the insertion point of BB2, and diagonally extending toward the anterior of the cell, also never merging with the apical complex.

#### 2.3.2. *Colpodella gonderi*

*Colpodella gonderi* is a free-living heterotrophic flagellate and is known to prey on a variety of ciliates [49,54]. Its two flagella emerge from the subapical region with two basal bodies forming a right angle. The basal bodies are noticeably shorter than those of dinoflagellates and perkinsids. They are interconnected with striated connective material (“interkinetosomal desmose” in the original article). There are at least two additional fibrous structures between the anterior basal body (BB2) and the striated fiber (figure 28 in [49]).

There are two microtubular roots. The numbering proposed here is interpreted from the original micrograph and in comparison with *C. vorax* [48], and has to be tested. The first one is perhaps R1 (“F1”), located on the left side of the posterior basal body (BB1). This root extends from BB1 at an approximately 60° angle, and does not seem to be directly connected to BB1, but instead attaches to the striated connective material at the proximal end.

The second root (“F2”) is a multi-microtubular (at least four) root that runs along the dorsal side of the anterior flagellar pit, and it is not clear if this is R3 or R4. The anterior flagellar pit seems to have a fibrous lining (figures 19, 28 and 33 in [49]).

No additional microtubule resembling the “oblique root” in *C. vorax* has been reported in *C. gonderi*.

#### 2.3.3. *Colpodella perforans*

*Colpodella perforans* is a free-living predatory flagellate feeding on other protists such as *Chilomonas paramecium* [52]. In light microscopy, *C. perforans* resembles other colpodellids, including *C. vorax* and *C. gonderi*, but its ultrastructural features suggests it may be closer to the dinoflagellates and perkinsids.

*C. perforans* has two flagella inserted sub-apically through separate flagellar pits. The basal bodies are arranged at right angles, or at a slightly acute angle. Neither of the basal bodies is as short as is seen in *C. vorax* and *C. gonderi.* The basal bodies are connected to one another by fibrous connective material. There seems to be at least two microtubular flagellar roots: an R1 that runs longitudinally from the posterior basal body (BB1), and an R4 with a transverse striated fiber emerging from the anterior basal body (BB2). There is another multi-microtubular structure, though it is unclear if it is homologous to R3 of dinoflagellates and perkinsids, or to the by-passing microtubules found in dinoflagellates, perkinsids and *C. vorax*.

## 3. Character Evolution

**Figure 6:**
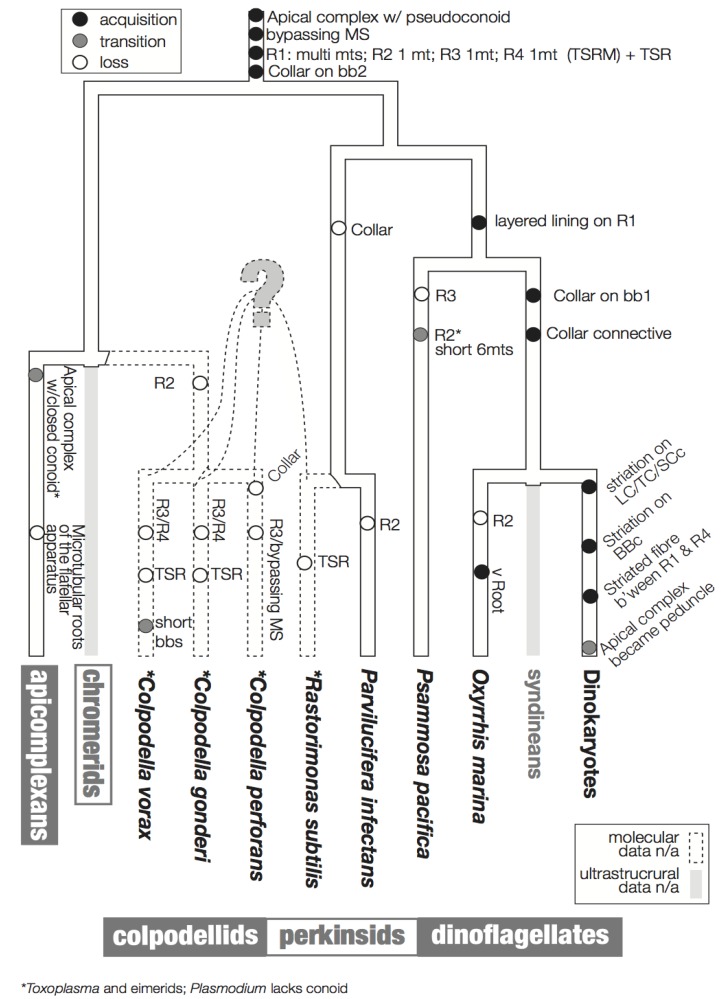
Flagellar apparatus character evolution. Character evolution of the flagellar apparatus of the dinoflagellates and the related lineages, perkinsids, colpodellids, and apicomplexans. The backbone tree topology is based on SSU rDNA [8,47]. The phylogenetic positions of *R. subtilis*, *C. gondi*, *C. perforans*, *C. vorans* are drawn with dotted lines as they are speculative due to a lack of molecular sequence data. Similarly, there is no ultrastructural information of the flagellar apparatus of syndineans, *Chromera*, and *Vitrella*, which are indicated by grey lines. The acquisition of a feature is indicated by a black circle, the transition of a feature is indicated by a grey circle, and the loss of a feature is indicated by an empty circle.

Figure 6 summarizes a proposed scheme for the evolution of these characters imposed on a hypothetical tree that is based primarily on molecular data. The hypothesized phylogenetic positions of *Rastrimonas subtilis*, *Colpodella perforans*, *C. gonderi, C. vorax* are all speculative due to the lack of the molecular sequences from these species, and are based on the positions of their inferred relatives where molecular data are known (*i.e.*, *Perkinsus*, *Parvilucifera* and *Colpodella*).

### 3.1. Microtubular Roots

In Ciliophora, a sister of the dinoflagellates and the related lineages discussed here, a standard unit of the flagellar apparatus includes two basal bodies and four microtubular roots [30,55]. Similarly, most flagellar apparatus of most dinoflagellates contains four microtubular roots, namely, R1 and R2 associated with basal body 1 (bb1; longitudinal basal body), and R3 and R4 associated with basal body 2 (BB2; transverse basal body). R1 and R4 are the most common roots that are universally present in all the known dinoflagellates and relatives. R1 is a multi-microtubular root with a layered fibrous lining, which is connected directly or indirectly via an additional fiber to BB1. Interestingly, in most dinokaryotes, R1 is also connected to transverse striated root (TSR) accompanying R4 via a connective fiber with the striation of a different pitch. This striated connective fiber between R1 and TSR is sometimes the only structure that connects BB1 and BB2 [56]. A structure resembling this striated connective fiber is observed between R1 and BB2 in *O. marina*. Analogous structures are missing from *Psammosa*, perkinsids or colpodellids.

R4 is a single microtubular root in known dinoflagellates and their relatives, except *R. subtilis* [43] and *C. vorax* [48]. In dinoflagellates (*i.e.*, dinokaryotes, *O. marina* and *P. pacifica*), *Pa. infectans*, and *C. perforans*, R4 is accompanied by the TSR. In *R. subtilis* R4 is still accompanied by a fibrous structure, though the fiber is not striated. 

R3 exhibits the most diversity across lineages. In dinokaryotes it is composed of a single microtubule with a few microtubules secondarily emerging from it (the transverse microtubular root extension; TMRE). In *O. marina* and *Pa. infectans* R3 is a single microtubule, but without TMRE. In *R. subtilis* R3 is composed of 2–3 microtubules. 

R2 is the simplest microtubular root, composed of only a single microtubule, without additional fibers or other structure in dinoflagellates. It is often missing, though this may be because R2 is hard to recognize because of its simplicity. The *Rastrimonas* R2 is exceptional in that it has two microtubules. Another exception is *P. pacifica*, which possesses a very short 6-microtubular root at the position of R2. It is not clear if the microtubules are homologous to R2 in other lineages, or rather to other multi-microtubular structures, such as ventral ridge root reported in *O. marina*.

### 3.2. Basal Body Collars and Fibrous Connective Structures

The striated collars and connective structures (the TSC-LSC-SCc complex) around the longitudinal and transverse basal bodies are unique components of the dinoflagellate flagellar apparatus. The “collars” are already present in basal lineages such as *Oxyrrhis* and *Psammosa*, *C. vorax*, and possibly in *C. gonderii*. The collars in the early branching lineages do not have the peculiar striation seen in the dinokaryotes. *O. marina* retains both collars on both basal bodies and the fibrous connective structures without striation. The collars in *O. marina* are ring-shaped and incomplete, surrounding only the ventral side of the basal bodies. *P. pacifica*, *C. vorax* and *C. gonderi* retain a collar only on the transverse basal body (BB2), which is speculated to be the ancestral state of the collars [41,48,49]. It is interesting that the collar first existed on the younger basal body (BB2; transverse basal body) and extended to the older basal body (BB1; longitudinal basal body) during the early evolution of dinoflagellates.

The collars and associated connective materials contain centrin in some dinoflagellates; namely, *Akashiwo sanguinea*, W*oloszynskia pascheri*, and *O. marina*. [29,40,57]. Centrin is one of the basic components of the eukaryotic cytoskeleton, and forms contractile fibers involved in multiple roles such as organelle segregation and cell motility [57,58]. The function of the collars and connective material is not known, although it is speculated to have a contractile function in order to change the orientation of the flagella, as is demonstrated in green algae [57]. It is also noteworthy that in some dinoflagellates, the TSC-LSC-SCc complex is connected to the microtubular strand of the peduncle, and may have an additional function to control the peduncle upon myzocytosis.

### 3.3. Is the Dinoflagellate Peduncle Homologous to the Apical Complex?

Dinozoa, Apicomplexa, and their associated lineages form a higher taxonomic entity called Myzozoa [6]. Myzozoan protists share myzocytosis as a synapomorphy. Myzocytosis was originally described as a mode of feeding in a dinoflagellate [59], where the predator penetrated a prey cell with a tube, through which it ingested the prey cytoplasm. This myzocytosis is mediated by the peduncle, but now several predators (e.g., colpodellids and probably *Psammosa*) have been found to use their apical complex to mediate the same feeding behavior. In the parasitic apicomplexans and perkinsids, the parasite injects itself into the host using the apical complex. The apical complex was first observed in *T. gondi* [60] and intensively studied in several apicomplexan parasites from the late 1950s through to the 1970s [61,62,63]. The apical complex of the apicomplexan parasites is composed of a microtubular conoid of closed truncated shape with a terminal ring at the apex; rhoptries, a type of electron dense vesicles of rhomboid shape with a narrow anterior neck and a wider posterior end; micronemes, a second type of electron dense vesicles; and dense granules, a third type of spherical vesicles that are larger than micronemes and contain electron dense materials [64]. The apical complexes later discovered in the related lineages, colpodellids, chromerids, perkinsids, and *Psammosa,* are similar, but have several noteworthy differences. These lineages possess what is referred to as the “archetype” apical complex, consisting of an open-sided conoid, or pseudoconoid [16], and a wide range of morphological diversity of vesicular components [6,42,43,44,46,48,50,51,61,62,65,66,67,68]. 

Interestingly, although they share myzocytosis, dinoflagellates lack a structure readily recognizable as an apical complex. The distribution of apical complexes indicates that the ancestor of dinoflagellates did possess one, so it must have either been lost or changed in morphology so much as to be nearly unrecognizable. Based on its function [59], one hypothesis is that the peduncle is homologous to the apical complex [6,42], however, there has been no direct evidence linking the two structures until recently [8,41]. 

Such evidence now comes from the cytoskeleton of *Psammosa*, one of the ancient lineages at the base of dinoflagellate [8]. *Psammosa* is a rare example within Myzozoa of a cell that possesses both an apical complex and flagellar apparatus at the same time throughout the vegetative stage of its life cycle. Both structures have now been reconstructed and found to be connected to one another via the bypassing microtubular strands [41]. Very similar microtubular strands are found in many dinoflagellates, and their characteristics, especially the spatial relation to the flagellar apparatus, are well documented [31,33,37,69,70,71,72,73,74,75,76,77]. As noted above, comparable bypassing microtubules are not only found in dinoflagellates, but also in perkinsids and colpodellids. However, the connection between the flagellar apparatus, the apical complex and the bypassing microtubule strand has not been observed, partly because the apical complex and flagellar roots have not been reconstructed together. This is likely because they do not co-exist in the best studied lineages. In the apicomplexans and in the parasitic perkinsids the flagellar apparatus and the apical complex is only found in zoospores, and may be morphologically reduced. The colpodellid flagellar apparatus also seems to be reduced, missing some microtubular roots (R2, and R3 or R4) compared to the dinoflagellate flagellar apparatus (summarized in Figure 6). In most colpodellids (and perkinsids), the zoospore loses its flagella and encysts to form four or more daughter cells (palintome) during division, which could have led to the reduction of the flagellar apparatus altogether. On the other hand, in dinoflagellates including *Oxyrrhis* and *Psammosa,* the flagellar apparatus is simply duplicated and inherited to the two daughter cells via the diagonal cell division. Another factor would be a technical development in microscopy, especially application of serial TEM tomography, which is a powerful new way to investigate and reconstruct three-dimensional structures in the cell. 

Both canonical and archetypical apical complexes contain secretory organelles, *i.e.*, rhoptries, micronemes, and dense granules [78]. In dinoflagellates, some elongated electron dense vesicles, morphologically similar to rhoptries or micronemes, are known to be associated with the peduncle (reviewed in [31]). The possible homology of these secretory organelles is of great interest, but also extremely difficult to assess. Rhoptries, micronemes, and elongated electron opaque vesicles are found across various lineages of the apicomplexans, colpodellids, perkinsids, and dinoflagellates, but there is generally little data on their possible function. With the current explosion of molecular data from apicomplexan parasites, as well as the growing number of genome projects, surveys, and transcriptomes from related lineages, the tools and information to tackle this very important question are beginning to fall into place. The key to this will be determining if the vesicular components retain a detectable signal of homology through the proteins they share.

At the same time, recent history has done much to underscore the importance of investing time and effort in the discovery of new organisms that would bridge known lineages. Recent analyses suggest a greater diversity of perkinsids, colpodellids and their relatives than we have previously recognized [15,19,47]. Each of these lineages has the potential to answer questions about the evolutionary history of dinoflagellates, apicomplexans and the related lineages, but only once they are characterized at the molecular and cellular levels.
